# Enhanced Immune Response Improves Resistance to Cadmium Stress in Triploid Crucian Carp

**DOI:** 10.3389/fphys.2021.666363

**Published:** 2021-06-04

**Authors:** Wen-bin Liu, Min-meng Wang, Liu-ye Dai, Sheng-hua Dong, Xiu-dan Yuan, Shu-li Yuan, Yi Tang, Jin-hui Liu, Liang-yue Peng, Ya-mei Xiao

**Affiliations:** ^1^State Key Laboratory of Developmental Biology of Freshwater Fish, Hunan Normal University, Changsha, China; ^2^College of Life Sciences, Hunan Normal University, Changsha, China

**Keywords:** cadmium, triploid crucian carp, immune response, liver function, transcriptome

## Abstract

Previous research has indicated that triploid crucian carp (3n fish) have preferential resistance to cadmium (Cd) compared to *Carassius auratas red var*. (2n fish). In this article, comparative research is further conducted between the 2n and 3n fish in terms of the immune response to Cd-induced stress. Exposure to 9 mg/L Cd for 96 h changed the hepatic function indexes remarkably in the 2n fish, but not in the 3n fish. In the serum of Cd-treated 2n fish, the levels of alanine amino transferase, aspartate aminotransferase, adenosine deaminase, and total bilirubin significantly increased, while the levels of total protein, albumin, lysozyme, and anti-superoxide anion radicals decreased demonstrating hepatotoxicity. By analysis of transcriptome profiles, many immune-related pathways were found to be involved in the response of 3n fish to the Cd-induced stress. Expression levels of the immune genes, including the interleukin genes, tumor necrosis factor super family member genes, chemokine gene, toll-like receptor gene, and inflammatory marker cyclooxygenase 2 gene were significantly enhanced in the hepatopancreas of the Cd-treated 3n fish. In contrast, the expression levels of these genes decreased in the 2n fish. This research provides a theoretical basis for polyploid fish breeding and is helpful for the ecological restoration of water due to pollution.

## Introduction

Polyploidy has been generated in many fish species (Cherfas et al., 1994; Bramick et al., 1995; Hamasak et al., 2013; Li et al., 2021). Compared with diploid (2n) fish, triploid (3n) fish often have superior phenotypic characteristics, such as an enhanced growth rate, lower mortality rate, and greater stress tolerance (Leggatt and Iwama, 2003; Seehausen, 2004; Nell and Perkins, 2005; Liu et al., 2016; Tao et al., 2018; Wang et al., 2020). In China, researchers generated a hermaphroditic allotetraploid hybrid by hybridizing female *Carassius auratas* with male *Cyprinus carpio* (Liu et al., 2004). By mating male allotetraploids with female *C. auratas*, they produced a strain of 3n crucian carp (named Xiangyunji) (Liu et al., 2004). This strain has a number of enhanced traits, such as fast growth, good flesh quality, and sterility, which are beneficial to ecological security and large-scale farming (Liu et al., 2004; Liu et al., 2018).

Cadmium (Cd) is toxic to aquatic organisms, and it can have long-term adverse effects on the aquatic environment (Rajan et al., 1995; Čelechovská et al., 2007; Vinodhini and Narayanan, 2008; Chen et al., 2013; Yuan et al., 2017; Liu et al., 2018). In animals, Cd is mainly enriched in the liver and kidney (Dudley et al., 1982; Thijssen et al., 2007; Włostowski et al., 2008; Elboshy et al., 2015). As a multifunctional organ, the liver is involved in metabolism and detoxification and also contains a large number of immune-related cells, such as Kupffer cells, macrophages, neutrophils, and lymphocytes (Racanelli and Rehermann, 2004; Nemeth et al., 2009; Yang et al., 2018). In fish, Cd exposure causes histopathological changes in hepatopancreas tissue (Sövényi and Szakolczai, 1993; Selvanathan et al., 2013; Liu et al., 2018). Wang et al. (2019) reported that acute Cd exposure had negative effects on stress defense, immunity, and metal transport systems in the hepatopancreas of zebrafish (*Danio rerio*) and grass carp (*Ctenopharyngodon idella*). Importantly, triploid crucian carp (3n fish) are preferentially resistant to Cd compared to *Carassius auratas red var.* (2n fish) (Liu et al., 2018). Previous reports also showed that under Cd stress, mortality and abnormality rates in 3n fish were lower than those of 2n strains, possibly due to their oxidative and endoplasmic reticulum stress responses.

In this study, we compared the immune response of 2n and 3n fish exposed to Cd-induced stress. Specifically, we assessed changes in hepatopancreas functions in the 2n and 3n fish after Cd exposure, and we evaluated whether 3n crucian carp had an enhanced immune response to Cd stress. The results of this study are valuable to polyploid fish breeding.

## Materials and Methods

### Ethics Statement

The animal study was reviewed and approved by the Animal Ethical Review Committee, Hunan Normal University, Changsha, China.

### Sample Preparation

All experiments followed the guidelines of the Animal Ethical Review Committee of Hunan Normal University, Changsha, China. Ten-month-old *C. auratus red var*. (2n fish) and Xiangyunji fish (3n fish) raised at the Engineering Center of Polyploidy Fish Breeding of the National Education Ministry located at Hunan Normal University were obtained for use in this study. The ploidy level of each sample was confirmed by a flow cytometer assay (Fu et al., 2020).

Fish were cultured in aerated water at 22°C without feeding for 1 week. The Cd exposure concentration was based on our previous 96-h experiments in which we identified the LC50 to be 9.0 mg/L for 2n fish and 15.0 mg/L for 3n fish (Gui, 2017). The Cd-treated 2n and 3n groups were incubated at 22°C for 96 h in CdCl_2_⋅2.5H_2_O solution (9 mg/L Cd^2+^, Tianjin Kermel Chemical Reagent Co., Ltd., Tianjin, China), and the corresponding 2n and 3n control groups were cultured in aerated water for 96 h at 22°C. In this study, each group consisted of seven fish cultured in a plastic tank containing ∼150 L of water. Fish were not fed, and dead fish were counted and promptly removed.

Fish were anesthetized with 100 mg/L of MS-222 (Sigma–Aldrich Co., Ltd., Shanghai, China) before dissection. For each subsequent test, we randomly selected three fish from each of the three groups, so as to form a sample with the same type of nine fish. Venous blood was taken from the caudal, and the serum was separated by centrifugation at 3,500 rpm for 15 min (Christian et al., 2018). Each serum sample was divided into two portions. The first was used to measure biochemical indicators, and the second was stored at −20°C for immunological assays. Additionally, pieces of the hepatopancreas were immediately frozen in liquid nitrogen and then preserved at –80°C for measurement of malondialdehyde (MDA) content and for mRNA sequencing and quantitative real-time polymerase chain reaction (qRT-PCR).

### Hepatopancreas Function Indices

The following hepatopancreas function indices in the serum were measured using an ADVIA2400 Automatic Biochemistry Analyzer (Siemens, Munich, Germany) and commercial kits (Beijing Solarbio Science and Technology Co., Ltd., Beijing, China) according to the manufacturer’s instructions: alanine aminotransferase (ALT), aspartate aminotransferase (AST), alkaline phosphatase (ALP), adenosine deaminase (ADA), total bilirubin (T-Bil), total protein (TP), albumin (ALB), and globulin (GLB). The activities of ALT, AST, ALP, and ADA were reported as U/L, the T-Bil content was expressed in μmol/L, and the levels of TP, ALB, and GLB were reported in g/L.

The level of MDA in the hepatopancreas tissue was measured using an MDA kit (Beijing Solarbio Science and Technology Co., Ltd., Beijing, China). The MDA content was determined based on the hepatopancreas quality, which was measured using a microplate reader (Chen et al., 2019). MDA content (reported as nmol/g tissue) was calculated by 5 × [6.45 × (A532-A600)−1.29 × A450]/0.1, where A450, A532, and A600 represented the absorbance of each sample at 450, 532, and 600 nm, respectively.

### Lysozyme Level and Anti-Superoxide Anion Radical Activity Analysis

Lysozyme (LZM) level in the serum and superoxide anion radical scavenging activities were measured using an LZM ELISA Kit and Inhibition and Produce Superoxide Anion Assay Kit (colorimetric method), respectively (Nanjing Jiancheng Biotechnology Co., Ltd., Nanjing, China) (Fang et al., 2012; Tang et al., 2014). Briefly, the LZM content (reported as μg/mL) was measured using a spectrophotometer at 530 nm and calculated by the following formula: [(UT_15_- OT_15_)/(ST_15__–_OT_15_)] × 200 U/L × dilution ratio, where T_15_ is the transmittance after 15 min at 37°C in a water bath and OT_15,_ UT_15_, and ST_15_ are the light transmittance values of the blank, test, and standard sample, respectively, after 15 min in the water bath. ASOR activity (reported as U/L) was measured by spectrophotometric analysis. It is based on the absorbance (OD) of each tube after zero adjustment with double distilled water at 550 nm. The value was calculated by the following formula: [(OD_blank_−OD_test_)/(OD_blank_−OD_standard_)] × 0.15 × 1,000 × dilution ratio.

### Transcriptome Data

The mRNA sequencing (seq) data for hepatopancreas tissues of the 3n fish and Cd-treated 3n fish were obtained from the NCBI SRA database (SRR8735277, SRR8735278, SRR13299805, SRR13299804) (Liu et al., 2018). Fragments per kb per million reads was used to calculate the gene expression levels of the two groups of fish. Differentially expressed genes (DEGs) were identified by two criteria:/log_2_ fold change (FC)/ > 1 and *p* < 0.05. To more intuitively reflect the difference and significance of DEG expression before and after Cd treatment, column and volcano plots were created using Excel and GraphPad Prism 7.0 software, respectively.

### qRT-PCR

We used qRT-PCR to examine the relative gene expression in hepatopancreas tissues of 2n and 3n fish. Total RNA was extracted using RNAiso Plus reagent (Takara, Bio., Beijing). RNA quality and purity were assessed by agarose gel electrophoresis and spectrophotometric analysis. One microgram from each RNA sample was used to synthesize cDNA using the Prime Script^TM^ RT Reagent Kit with gDNA Eraser (Takara). Primers were designed using Primer Premier 5.0 software (Table 1). The housekeeping gene β*-actin* was used as the reference gene. The reaction mixtures were added to 96-well plates and incubated in the Prism 7500 Sequence Detection 140 System (Applied Biosystems, Foster City, CA, United States). The reaction mix contained 1 μL of cDNA, 5 μL of 2 × SYBR Green qPCR Master Mix (Biotools, Jupiter, FL, United States), 0.5 μL of forward primer, 0.5 μL of reverse primer, and 3 μL of diethylpyrocarbonate water. The PCR program was as follows: 1 cycle of 50°C for 2 min and 95°C for 10 min; 40 cycles of 95°C for 15 s and 60°C for 1 min; and dissociation curve analysis (60–95°C) (Mo et al., 2019). The relative expression ratio of target genes vs. the β*-actin* gene was calculated using the 2^–△△Ct^ method.

**TABLE 1:** Primer sequences used for qRT-PCR.

**Gene ID**	**Gene name**	**Primer pair (5′–3′)**
comp249580_c0_seq1	β*-actin*	F: ATACTCCTGCTTGCTAATCCAC
		R: ATGTACCCTGGCATTGCT
comp234816_c0_seq2	*il1*β	F: TGTCTTCGCATCCTCACAGC
		R: GACGCTCTTCGATCACATTCT
comp237000_c0_seq9	*il6*	F: CGATCCTGTTCAACTTCACC
		R: CTGCTCTGAATGAACTCTCTG
comp256289_c0_seq2	*tnfsf12*	F: CTCATGCCACAGAATCAGGT
		R: TTGACCCAGCAAATCAGTCG
comp247992_c1_seq2	*tnfrsf13*β	F: CCAGCATCTCAACATAGTCCTG
		R: GGCCTTCAAAGTGTGTTGTG
comp224930_c1_seq3	*ccl4*	F: CCAGTAAAGCAGGTTGAGAG
		R: TCACATCTTATTCGCTGTCC
comp247841_c0_seq1	*cox2*	F: CAGTACCAGAACCGCATCG
		R: GTTACGTCCACCAGCAACC
comp233981_c0_seq3	*tlr4*	F: TCCAGCTATGATGAAGTCTG
		R: CGACCACAATGATTTTACGA
comp235303_c3_seq1	*hsp70*	F: CTGATTCTGCCACACACGTT
		R: GCAAGTTTGGAAGCAGTACAG
comp254412_c0_seq1	*fos*	F: CAACCCAAACCCTTACCCT
		R: GCAGCAGCCATCTTATTCC

### Statistical Analysis

Statistical analysis was performed using the paired *t*-test for comparison of two groups (Rosario et al., 2014; Zheng et al., 2016; Liu et al., 2018; Mo et al., 2019). For multiple testing, a Bonferroni *post hoc* test of *p-*values was performed. Data were expressed as mean ± standard error of the mean (SEM) of at least three independent experiments. A *p* < 0.05 was considered to be statistically significant.

## Results

### Effects of Cd on Hepatopancreas Function of 2n and 3n Crucian Carp

Prior to Cd exposure, there were no significant differences in the levels of ADA, T-Bil, GLB, and TP between the 2n and 3n fish. However, the ALT and ALB levels were higher and the AST and ALP were lower in the 3n fish compared to the 2n fish.

In comparison with the 2n control group, some hepatopancreas function indices changed significantly after 2n fish were exposed to Cd. The activities of ALT, AST, and ADA, and the T-Bil content increased by 99, 157, 197, and 111%, respectively (Table 2). However, there was no obvious difference in most index levels between control and Cd-treated 3n fish. The exceptions were ALT and ADA activities, which decreased by 31 and 61%, respectively (Table 2).

**TABLE 2:** Effects of Cd exposure on serum hepatopancreas function indices.

	**2n control**	**2n-treated**	**3n control**	**3n-treated**
ALT (U/L)	10.03 ± 1.79	19.97 ± 1.28**	47.95 ± 5.05	32.97 ± 5.45
AST (U/L)	235.70 ± 27.14	605.87 ± 42.49***	130.20 ± 9.00	131.03 ± 20.83
ALP (U/L)	70.17 ± 4.38	76.13 ± 4.46	42.75 ± 2.25	43.65 ± 2.25
ADA (U/L)	18.84 ± 3.05	55.98 ± 13.72*	20.77 ± 4.21	8.07 ± 0.12*
T-Bil (μmol/L)	1.26 ± 0.20	2.66 ± 0.32**	1.52 ± 0.49	1.65 ± 0.48
TP (g/L)	28.23 ± 1.47	24.47 ± 0.39*	35.37 ± 4.94	36.57 ± 6.42
ALB (g/L)	5.60 ± 0.36	4.70 ± 0.22*	13.23 ± 4.81	11.40 ± 3.76
GLB (g/L)	22.63 ± 1.22	19.78 ± 0.19*	22.13 ± 2.58	25.17 ± 2.67

Data are presented as mean ± SEM (n = 3 replicates). *Indicates significant difference between the control group and Cd-treated group (*p < 0.05, **p < 0.01, ***p < 0.001).

The activities of AST, ALP, and ADA, and the T-Bil content in the Cd-treated 2n fish were about 4.62, 1.74, 6.93, and 1.61 times higher than those of the Cd-treated 3n fish. In contrast, the levels of TP and ALB in the Cd-treated 3n fish were 1.49 and 2.42 times higher than those in the Cd-treated 2n fish.

### Changes in MDA Content in Hepatopancreas Tissue Under Cd Stress

**FIGURE 1:**
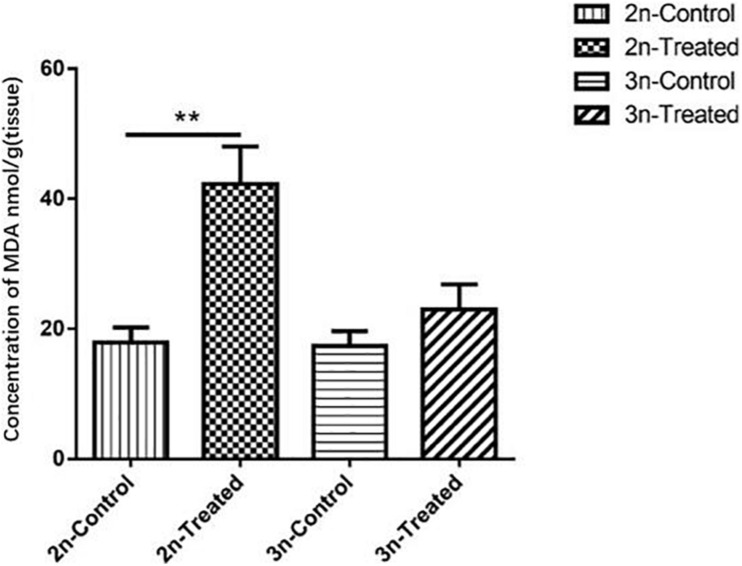
Changes of MDA concentration in the hepatopancreas of *C. auratus red var*. (2n) and triploid crucian carp (3n). *Indicates significant differences between the Cd-treated group and corresponding control group (**p < 0.01). Each bar represents the mean ± SD of three independent experiments.

MDA level is a marker of lipid peroxidation (Gaweł et al., 2004). Before Cd exposure, the MDA level in the hepatopancreas tissue did not differ significantly between the 2n and 3n fish. After Cd exposure, the MDA content was significantly higher (*p* < 0.01) in the 2n fish compared to their control group, but the MDA content of the Cd-treated 3n fish and their control group did not differ significantly (*p* > 0.05). The MDA content of Cd-treated 2n fish was 1.80 times higher than that of Cd-treated 3n fish (Figure 1).

### Effects of Cd Stress on Serum LZM and ASOR Activity

**FIGURE 2:**
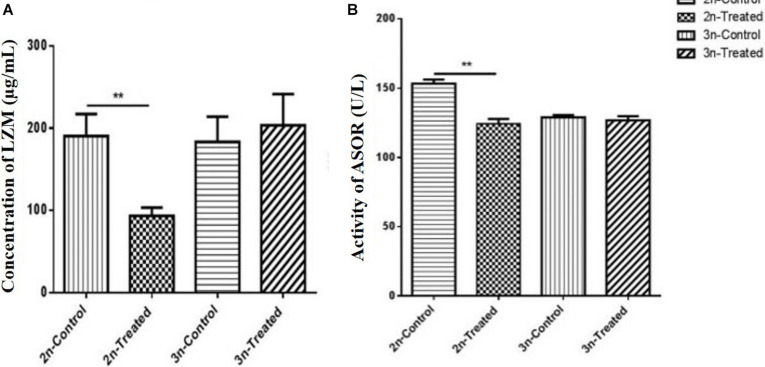
Effects of Cd exposure on the concentration of LZM and activity of ASOR in the hepatopancreas of 2n *C. auratus red var*. and 3n crucian carp. **(A)** Effect of Cd exposure on serum LZM content. **(B)** Effect of Cd exposure on serum ASOR activity. *Indicates significant difference between the Cd-treated group and corresponding control group (**p < 0.01). Each bar represents the mean ± SD of three independent experiments.

LZM is an immune-active substance that participates in the body’s non-specific immunity, and it is one of the basic defenses for immune regulation (Enis et al., 2019). Prior to Cd exposure, the serum LZM level did not differ between the 2n and 3n fish. Under Cd stress, however, the LZM content was significantly decreased in 2n fish relative to their control (*p* < 0.01), whereas there was no significant change in the 3n fish compared to their control (*p* > 0.05) (Figure 2A). The level of LZM in the Cd-treated 3n fish was 2.33 times higher than that in the Cd-treated 2n fish.

Prior to Cd exposure, ASOR activity of 2n fish was significantly higher than that of 3n fish (*p* < 0.01). ASOR activity was inhibited significantly in the Cd-treated 2n fish relative to their control (*p* < 0.01), but it remained unchanged in the Cd-treated 3n fish (*p* > 0.05) compared to the corresponding control (Figure 2B).

### Transcriptome Profiling Analysis of Cd Impacts on Hepatopancreas in Triploid Crucian Carp

**FIGURE 3:**
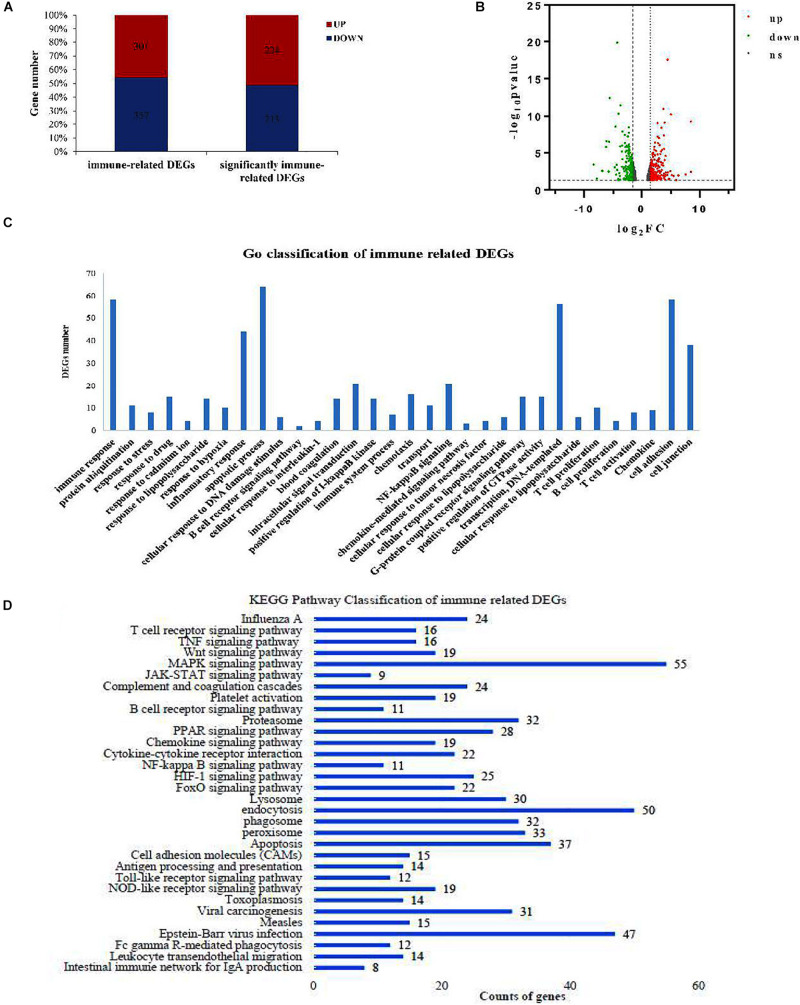
Transcriptome analysis of hepatopancreas tissue under Cd stress in 3n crucian carp. **(A)** Immune-related DEGs between the Cd-treated group and control 3n fish. **(B)** Volcano distribution of the immune-related DEGs (up represents log_2_FC ≥ 1.5, down represents log_2_FC ≤ –1.5, ns represents/log_2_FC/ < 1.5). **(C)** GO enrichment of the immune-related DEGs. **(D)** Kyoto Encyclopedia of Genes and Genomes (KEGG) pathway classification of immune-related DEGs.

In the hepatopancreas tissue of 3n fish, we found 658 genes that were differentially expressed between the control and Cd-treated groups, of which 301 were upregulated and 357 were downregulated (Figure 3A). Moreover, 437 immune-related DEGs were significant, with 224 upregulated and 213 downregulated genes. Figure 3B shows the volcano distribution of these immune-related DEGs.

The immune response, inflammatory response, and apoptosis were the main Gene Ontology (GO) items that were enriched (Figure 3C). These DEGs were mainly involved in 32 immune-related pathways, in which the MAPK pathway and endocytosis were significantly enriched (Figure 3D). Many important immune factors were significantly upregulated in the hepatopancreas of 3n fish after Cd treatment compared with their control group, such as signal transducers (*tlr4, jun, junb, fos, creb5, par2*), cytokines and cytokine receptors (*il6, tnfrsf5, tnfrsf13*, *tnfsf10, tnfsf12*), chemokines and chemokine receptors (*ccr9, ccl4, cxcr4, cxcr3*), heat shock proteins (*hsp70, hsp90a*), and the inflammatory inducible enzyme (*cox2)* (Table 3).

**TABLE 3:** Immune-related DEGs in the hepatopancreas of Cd-treated 3n crucian carp.

**KEGG_n**	**Gene name**	**3n-Cd-treated VS. 3n conControl**		**Note**
		**log_2_ fold change**	***p*-value**	
Cytokines and cytokine receptors	*il6*	4.69	3.49E-02	Interleukin 6
	*tnfrsf5*	2.57	2.32E-02	Tumor necrosis factor receptor superfamily member 5
	*tnfrsf13*	3.85	5.07E-08	Tumor necrosis factor receptor superfamily member 13B
	*tnfsf10*	1.42	3.73E-03	Tumor necrosis factor ligand superfamily member 10
	*tnfsf12*	2.20	1.47E-02	Tumor necrosis factor ligand superfamily member 12
Chemokines and chemokine receptors	*ccr9*	1.38	2.56E-02	C-C chemokine receptor type 9
	*ccl4*	2.17	8.74E-03	C-C motif chemokine 4
	*cxcr4*	1.16	4.36E-02	C-X-C chemokine receptor type 4
	*cxcr3*	2.38	3.46E-02	C-X-C chemokine receptor type 3
Signal transducers Heat shock protein	*tlr4*	2.19	7.02E-02	Toll-like receptor 4
	*jun*	1.59	2.28E-2	Transcription factor AP-1
	*junb*	1.57	3.18E-04	transcription factor jun-B
	*fos*	3.87	6.81E-03	Proto-oncogene protein c-fos
	*creb5*	3.94	8.66E-10	Cyclic AMP-responsive element-binding protein 5
	*par2*	3.74	1.08E-11	Coagulation factor II (thrombin) receptor-like 1
	*hsp70*	1.52	1.13E-02	Heat shock 70kDa protein
	*hsp90a*	3.19	2.25E-02	Heat shock protein 90kDa-alpha
Cell adhesion molecules (CAMs)	*nfasc*	4.02	3.81E-02	Neurofascin
	*b7h3*	3.23	3.54E-02	Immune costimulatory protein B7-H3
	*cd2*	1.90	4.87E-03	T-cell surface glycoprotein CD2
Inflammatory inducible enzyme	*cox2*	1.97	1.10E-02	Prostaglandin-endoperoxide synthase 2

### Expression Levels of Immune-Related Genes in the Hepatopancreas of Cd-Treated 2n and 3n Crucian Carp

**FIGURE 4:**
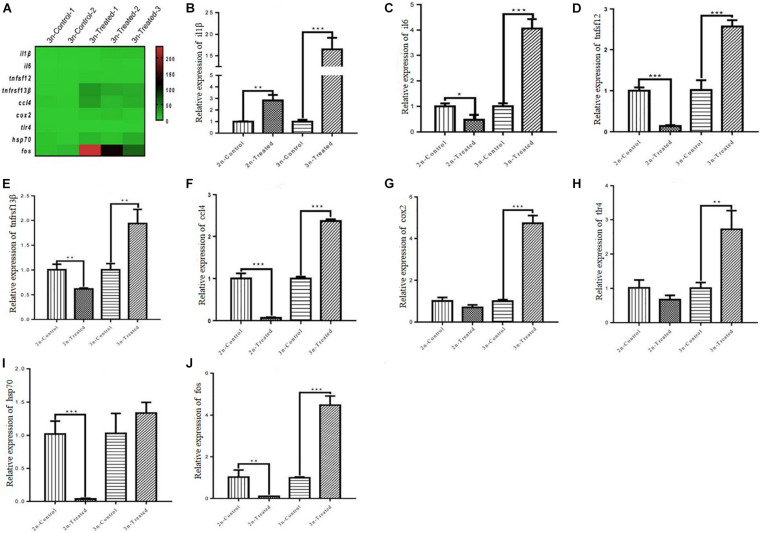
Expression levels of the nine genes detected by mRNA-seq and qRT-PCR in hepatopancreas tissues. **(A)** Heatmap of the expression distribution of the nine genes as detected by mRNA-seq in hepatopancreas tissue of 3n crucian carp. **(B–J)** Expression levels of the nine genes detected by qRT-PCR in hepatopancreas tissues of 2n and 3n crucian carp. *Indicates significant difference between the Cd-treated group and corresponding control group (*p < 0.05, **p < 0.01, ***p < 0.001).

To further understand the immune response of crucian carp exposed to Cd, nine important immune-related DEGs were selected from the hepatopancreas transcriptome of 3n fish (*il1*β, *il6*, *tnfsf12*, *tnfrsf13*β, *ccl4*, *tlr4, hsp70*, *cox2*, and *fos*) (Figure 4A). The qRT-PCR analysis revealed significant differences in gene expression levels between 2n and 3n fish under Cd stress. Compared with their control group, Cd-treated 2n fish exhibited significantly upregulated mRNA expression of *il1*β but downregulated expression of *il6, tnfsf12, tnfrsf13*β, *ccl4, cox2, tlr4, hsp70*, and *fos.* In the Cd-treated 3n fish, the mRNA expression levels of all nine genes were upregulated relative to the control (Figures 4B–J).

## Discussion

After fish were exposed to Cd, many hepatopancreas function indices (e.g., the activities of ALT, AST, and ADA and the content of T-Bil) in the serum were higher in 2n *C. auratus red var*. than in the 3n crucian carp. The serum contains various humoral immune components, and it can accurately reflect the immune level of the body (Banavreh et al., 2019; Devi et al., 2019). In mammals, ALT and AST are two important transaminases that are sensitive indicators of liver cell injury (Cheng et al., 2013). T-Bil and TP levels can also reflect liver damage (Hafez et al., 2018). ADA is a nucleic acid metabolic enzyme, and its activity has an important relationship with the immune activity of cells, which makes it a sensitive index of liver injury (Baldissera et al., 2018a). The level of ADA in serum is a useful marker for patients with liver cirrhosis complicated by refractory ascites in order to diagnose tuberculous peritonitis (Shimozuma et al., 2009). Similarly, ADA levels are associated with disease activation in patients with other autoimmune diseases, such as systemic lupus erythematosus, juvenile idiopathic arthritis, rheumatoid arthritis, and Still’s disease (Torgutalp et al., 2017). In grass carp, Feng et al. (2004) found that parts of hepatocytes were lytic and necrotic after Cd poisoning, and the AST and ALP activities in blood plasma of experimental groups were significantly higher than those in the corresponding control group, whereas the TP level was significantly lower. Baldissera et al. (2018b) proposed that downregulation of serum ADA activity exerted an anti-inflammatory effect that contributed to restricting the inflammatory process in silver catfish. Our results also indicated that Cd exposure led to acute damage to the hepatopancreas in 2n *C. auratus red var*. These results are consistent with previous hepatocellular histological observations (Liu et al., 2018). Thus, we concluded that 2n *C. auratus red var*. was more vulnerable to Cd exposure than 3n crucian carp based on the hepatopancreas function indices.

In organisms, MDA causes the cross-linking polymerization of proteins and nucleic acids, and it can aggravate membrane damage. Increased production of MDA reflects the degree of oxidative damage in the body (Shi et al., 2019; Wang et al., 2019; Gu et al., 2020). On the other hand, LZM can cause bacterial lysis that actives phagocytes and complement systems, which act as opsonins in mucus, serum, and other body fluids. Thus, LZM can be an important non-specific immune defense protein in exogenous toxic resistance in fish (Gou et al., 2018; Jawahar et al., 2018; Enis et al., 2019). Additionally, antioxidant indicators are often used to indicate the body’s immune function (Gou et al., 2018; Wen et al., 2018). Oxidative stress in the body caused by exposure to Cd may be an important reason for immune function imbalance (Biller-Takahashi et al., 2015; Cobbina et al., 2015). We found that the content of MDA increased significantly in Cd-exposed 2n *C. auratus red var*. but not in 3n crucian carp (Figure 1). The Cd exposure resulted in significant decreases in levels of serum LZM and ASOR in *C. auratus red var*. relative to their control, but no significant change in 3n crucian carp was detected (Figure 2). Therefore, our results suggested that Cd exposure caused excessive oxidative stress in 2n but not in 3n crucian carp.

Fish immunity mainly involves the innate immune response, which is composed of bactericidal substances (such as LZM), interleukins (ILs), tumor necrosis factor, chemokines, lectins, and other non-specific immune factors (Modanloo et al., 2017; Vazirzadeh et al., 2017; Xiao et al., 2017). ILs are cytokines that can be synthesized by many kinds of immune cells, and they mediate the activation, proliferation, and differentiation of different immune cells (Ye and Zeng, 2019). A variety of IL families have been identified in fish (Pan et al., 2005), and IL-1β and IL-6 have been shown to induce the expression of various inflammatory factors to stimulate the immune response (Rajeshkumar et al., 2017; Devi et al., 2019). Chemokines are key regulators of the immune response. They activate chemotactic leukocytes to the infected or damaged site and also regulate the differentiation and immune response of some recruited cells (Alejo and Tafalla, 2011; Zhang et al., 2017). Our hepatopancreas transcriptome data revealed that many immune-related pathways were involved in the immune regulation of 3n crucian carp exposed Cd stress (Figure 3). In the hepatopancreas tissue of 3n crucian carp, Cd exposure significantly upregulated the expression of *il1*β, *il6, tnfsf12, tnfrsf13*β, *ccl4, tlr, hsp70, cox2, and fos* (Figure 4). In contrast, the expression levels of these genes in the Cd-treated 2n fish decreased significantly. These results suggest that the better immunity of 3n crucian carp compared to 2n fish might alleviate the pathological changes to the hepatopancreas caused by exposure to Cd.

Distant hybridization is an important technique for generating polyploidy, and it can lead to an altered genotype and phenotype of the offspring (Liu et al., 2016; Ren et al., 2019). Parsons et al. (1986) found that 3n rainbow trout × coho salmon hybrids showed increased resistance to infectious hematopoietic necrosis virus, and Fu et al. (2020) reported that the artificial induction of triploidy may improve the survival rate of distant hybrids. Xiao et al. (2019) showed that 3n crucian carp displayed stronger disease resistance compared with their parents and proposed that this effect occurred through the mitochondrial antiviral-signaling protein. Moreover, in general, the triploid fish were considered to be sterile (Krisfalusi et al., 2000; Piferrer et al., 2009; Qin et al., 2015; Huang et al., 2016; Hu et al., 2018), which not only ensures ecological security, but also brings the advantage of rapid growth (Lincoln and Scott, 1983; Guo et al., 2004; Yan et al., 2005; Liu et al., 2006, 2007; Hu et al., 2012). Interestingly, there are also some fertile fish in triploids (Kavumpurath and Pandian, 1990; Zhang and Arai, 1999b; Xiao et al., 2011; Peng et al., 2020). In this study, the triploid crucian carp has been used as experimental materials and it was found that improved resistance to cadmium stress in triploid crucian carp can be the result of enhanced immune activity. In future research, there are still some interesting issues to further address the effects of polyploidization on the development of fish, such as reproductive characteristics and stress resistance.

## Conclusion

Cd-induced toxicity is a classic model used to study environmental impacts on fish. In this study, we compared the changes in hepatopancreas functions induced by Cd exposure between 2n and 3n crucian carp. Our results confirmed that hepatopancreas functionality is the main target of Cd toxicity in fish and that 2n *C. auratus red var.* were more sensitive to Cd stress than 3n crucian carp. Our transcriptome analysis showed that immunity-related genes were involved in the response of crucian carp to Cd stress. These results helped explain why triploidy improved the resistance of fish to Cd stress, and can provide an important theoretical basis for polyploid fish breeding and the ecological restoration of polluted water.

## Data Availability Statement

The original contributions presented in the study are included in the article/supplementary material, further inquiries can be directed to the corresponding author/s.

## Ethics Statement

The animal study was reviewed and approved by the Animal Ethical Review Committee, Hunan Normal University, Changsha, China.

## Author Contributions

Y-MX, W-BL, and M-MW designed the experiments and organized and wrote the manuscript. W-BL, M-MW, L-YD, S-HD, X-DY, S-LY, and YT carried out the experiments. W-BL, M-MW, J-HL, L-YP, and Y-MX conducted the statistical analysis and wrote the discussion. All authors read the manuscript and agreed to list their names as coauthors.

## Conflict of Interest

The authors declare that the research was conducted in the absence of any commercial or financial relationships that could be construed as a potential conflict of interest.

## Abbreviations

ALT: alanine aminotransferase
AST: aspartate aminotransferase
ALP: alkaline phosphatase
ADA: adenosine deaminase
T-Bil: total bilirubin
TP: total protein
ALB: albumin
GLB: globulin
MDA: malondialdehyde
LZM: serum activities of lysozyme
ASOR: anti-superoxide anion radicals
ROS: reactive oxygen species
IL: interleukin
TNF: tumor necrosis factor
AP-1: transcription factor activating protein-1
SEM: stranded error of the mean
*il1* β: interleukin genes 1 β
*il6*: interleukin genes 6
*tnfsf12*: tumor necrosis factor (TNF) super family member genes 12
*tnfrsf13* β: tumor necrosis factor (TNF) superfamily member genes 13 β
*tlr4*: toll-like receptor gene
*ccl4*: chemokine gene
*hsp70*: heat shock protein 70 gene
*cox2*: inflammatory marker cyclooxygenase 2 gene
*fos*: transcription factor activating protein-1 member genes.
